# Comparison of Japanese and Indian intestinal microbiota shows diet-dependent interaction between bacteria and fungi

**DOI:** 10.1038/s41522-019-0110-9

**Published:** 2019-12-20

**Authors:** Siddhika Pareek, Takashi Kurakawa, Bhabatosh Das, Daisuke Motooka, Shuuichi Nakaya, Temsunaro Rongsen-Chandola, Nidhi Goyal, Hisako Kayama, Dylan Dodd, Ryu Okumura, Yuichi Maeda, Kosuke Fujimoto, Takuro Nii, Takao Ogawa, Tetsuya Iida, Nita Bhandari, Toshiyuki Kida, Shota Nakamura, G. Balakrish Nair, Kiyoshi Takeda

**Affiliations:** 1 Department of Microbiology and Immunology, Graduate School of Medicine, Osaka University, Suita, Osaka, 565-0871 Japan; 2 WPI Immunology Frontier Research Center, Osaka University, Suita, Osaka, 565-0871 Japan; 3 Core Research for Evolutional Science and Technology, Japan Agency for Medical Research and Development, Tokyo, 100-0004 Japan; 4 Molecular Genetics Laboratory, Center for Human Microbial Ecology, Translational Health Science and Technology Institute, Faridabad, 121001 India; 5 Department of Infection Metagenomics, Genome Information Research Center, Research Institute for Microbial Diseases, Osaka University, Osaka, 565-0871 Japan; 6 Global Applications Development Center, Shimadzu Corp, Kyoto, 604-8511 Japan; 7 Centre for Health Research and Development, Society for Applied Studies, New Delhi, 110016 India; 8 Department of Pathology, Stanford University School of Medicine, Stanford, CA 94305 USA; 9 Department of Respiratory Medicine and Clinical Immunology, Graduate School of Medicine, Osaka University, Osaka, 565-0871 Japan; 10 Department of Bacterial Infections, Research Institute for Microbial Diseases, Osaka University, Osaka, 565-0871 Japan; 11 Department of Applied Chemistry, Graduate School of Engineering, Osaka University, Osaka, 565-0871 Japan

**Keywords:** Symbiosis, Microbiome

## Abstract

The bacterial species living in the gut mediate many aspects of biological processes such as nutrition and activation of adaptive immunity. In addition, commensal fungi residing in the intestine also influence host health. Although the interaction of bacterium and fungus has been shown, its precise mechanism during colonization of the human intestine remains largely unknown. Here, we show interaction between bacterial and fungal species for utilization of dietary components driving their efficient growth in the intestine. Next generation sequencing of fecal samples from Japanese and Indian adults revealed differential patterns of bacterial and fungal composition. In particular, Indians, who consume more plant polysaccharides than Japanese, harbored increased numbers of *Prevotella* and *Candida*. *Candida* spp. showed strong growth responses to the plant polysaccharide arabinoxylan in vitro. Furthermore, the culture supernatants of *Candida* spp. grown with arabinoxylan promoted rapid proliferation of *Prevotella copri*. Arabinose was identified as a potential growth-inducing factor in the *Candida* culture supernatants. *Candida* spp. exhibited a growth response to xylose, but not to arabinose, whereas *P. copri* proliferated in response to both xylose and arabinose. *Candida* spp., but not *P. copri*, colonized the intestine of germ-free mice. However, *P. copri* successfully colonized mouse intestine already harboring *Candida*. These findings demonstrate a proof of concept that fungal members of gut microbiota can facilitate a colonization of the intestine by their bacterial counterparts, potentially mediated by a dietary metabolite.

## Introduction

The human gut bacterial community represents an enormous number of 10^14^ bacteria with more than 1000 different species and has diverse roles, such as maintaining immune homeostasis, freeing dietary nutrients for host absorption, and colonization resistance against pathogens.^1^ The gut bacterial composition varies immensely among individuals in response to intrinsic and extrinsic factors including genetic background, mode of delivery during childbirth, age, diet, and diseases.^2–4^ High throughput sequencing technologies have enabled comprehensive analyses of the human microbiome.^5^ Several studies investigating the composition of human microbiota have shown that environmental factors rather than host genetics play a crucial role in shaping the intestinal microbial ecosystem.^6–8^ Among these environmental factors, dietary components strongly influence the bacterial composition of the gut.^9^ Generally, plant-based diets are more prevalent in developing countries, whereas the intake of animal-derived products is higher in developed countries, as shown in the database of Food and Agriculture Organization of the United Nations (http://www.fao.org/faostat/en/home). The gut microbiota was clustered into three enterotypes, characterized by the abundance of *Bacteroides*, *Prevotella*, and *Ruminococcus*^10^ and long-term diet were suggested to influence these enterotype patterns across the populations. Several studies covering different populations worldwide have shown that consumption of animal-based diets and plant-based diets induces differential patterns of gut bacterial composition.^6,9,11–13^ However, these studies exploring the impact of intestinal microbes and metabolic health have essentially focused on bacteria in the intestine.^14,15^ Notably, fungal species have been reported to colonize as commensals in the gut of healthy humans and mice.^16,17^ Although they comprise less than 1% of the total gut microbial population,^10,18^ studies in murine models have shown its importance during alteration of the gut environment. For instance, *Candida albicans* is persistently present in a mouse model that develops allergic disorders and autoimmune diseases,^19^ and was shown to interact with bacteria during gastric colonization.^20^ The commensal bacteria prevent fungi from long-term colonization.^21^ During antibiotic recovery in the murine cecum, *C. albicans* was shown to promote the restoration of bacterial diversity.^22^ In addition, diet has been shown to modify the abundance of the fungal population as well as bacterial population in the gut.^18^ These findings, therefore, underline the necessity to examine the precise mechanism of interkingdom interactions to understand microbiome-mediated effects on host physiology.

In this study, we analyzed bacterial and fungal composition of Japanese and Indian fecal samples. Based on the dietary habitat questionnaire and their dominant microorganisms, we focused on metabolism of arabinoxylan, which is one of the major indigestible polysaccharides. We then analyzed the potential mechanism for interaction between gut bacterium and fungus both in vitro and in vivo. The results suggested a dietary metabolite-dependent interaction between fungi and bacteria, which promotes bacterial growth and colonization in the gut.

## Results

### Analyses of bacterial and fungal composition in Japanese and Indian feces

**Fig. 1:**
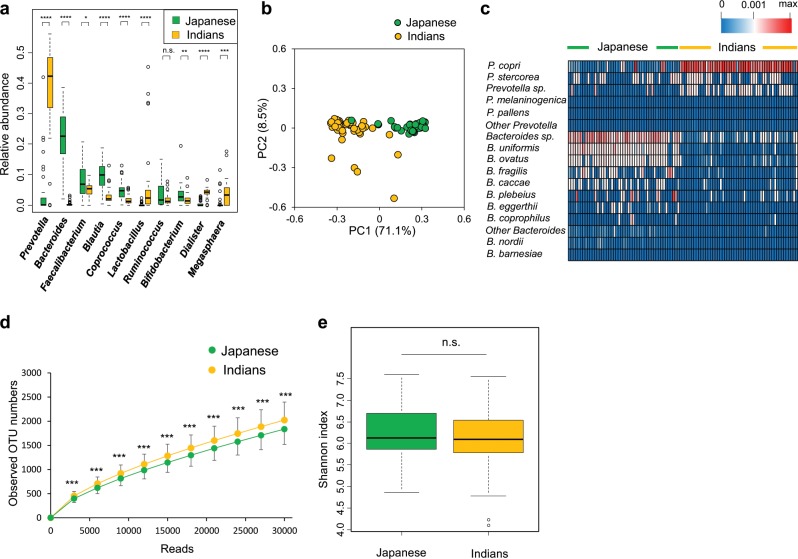
Comparison of fecal bacteria in healthy adults living in Japan and India. **a** Relative abundances of the major genera. **b** Principal component analysis of fecal bacteria at the genus level. **c** Heatmap representing the relative abundances of bacterial species in genus *Bacteroides* and *Prevotella*. **d** Rarefaction curves. **e** Shannon index. n.s. not significant; ****p < 0.0001, ***p < 0.001, **p < 0.01, *p < 0.05.

We compared the composition of fecal bacteria and fungi from two geographically distinct healthy adult populations living in Japan (*n* = 47) and India (*n* = 50) (Table 1). First, bacterial compositions were compared by 16S rRNA gene sequencing. Firmicutes, Bacteroidetes, Actinobacteria, and Proteobacteria were the four dominant bacterial phyla in both Japanese and Indian samples (Supplementary Fig. 1a, b). The ratios of Bacteroidetes to Firmicutes in individuals from India were markedly higher than those from Japan (Supplementary Fig. 1c, d). Genus level analysis showed that *Bacteroides* and *Prevotella* were the dominant bacterial genera in samples from Japan and India, respectively (Fig. 1a). Principal component analysis (PCA) based on gut bacterial composition showed distinct clustering of Indian and Japanese samples (Fig. 1b). Considering the genus *Prevotella*, individuals from India harbored *Prevotella copri* as the dominant bacterial species, followed by *Prevotella stercorea* and *Prevotella* sp., while these species were detected only in a small number of those from Japan (Fig. 1c). With respect to the genus *Bacteroides*, *Bacteroides* sp., *Bacteroides* *uniformis*, *Bacteroides ovatus*, and *Bacteroides fragilis* were prevalent in Japanese samples. Although higher numbers of operational taxonomic units (OTUs) were observed in samples from India than Japan, both groups showed similar Shannon diversity, which would be due to the high proportion of *Prevotella* in Indian samples (Fig. 1d, e). This observation is in accordance with previous reports on analysis of microbiota from populations consuming diets rich in plant-derived products.^6,7,23^

**Table 1:** Subjects information in this study.

Issues	Japanese	Indians	Statistical difference
District of residence	Osaka	Delhi	
Subject number	47	50	
Male (%)	54	53	
Age	28.8 ± 6.2	30.6 ± 6.1	
Animal exposure (%)	17	38	*
Cattle	0	20	**
Goat/Sheep	0	2	
Dog	2	4	
Cat	4	2	
Others (including no answer)	11	10	
Places of defecation (%)			
Private toilet at home	100	88	*
Public toilet	0	18	**
Open field	0	26	***
Food (frequency of ingestion in 1 week)			
Bread	5.6 ± 1.9	14.4 ± 4.3	****
Rice^a^	6.5 ± 1.8	4.2 ± 2.7	****
Maize	1.2 ± 1.9	0.4 ± 0.8	**
Soybean^b^	4.6 ± 3.4	0.1 ± 0.4	****
Yogurt	2.7 ± 2.3	2.2 ± 2.3	
Cheese	2.0 ± 1.8	0.1 ± 0.2	****
Butter	2.9 ± 2.7	1.9 ± 2.6	*
Milk	4.7 ± 3.7	4.6 ± 3.8	
Meat	4.2 ± 1.8	0.3 ± 0.8	***
Fish	2.5 ± 1.5	0.1 ± 0.4	****
Poultry	2.7 ± 1.7	0.4 ± 0.8	****
Eggs	4.2 ± 1.9	1.1 ± 2.1	****

^a^Includes roti

^b^Includes tofu and natto

*p < 0.05

**p < 0.01

***p < 0.001

****p < 0.0001

**Fig. 2:**
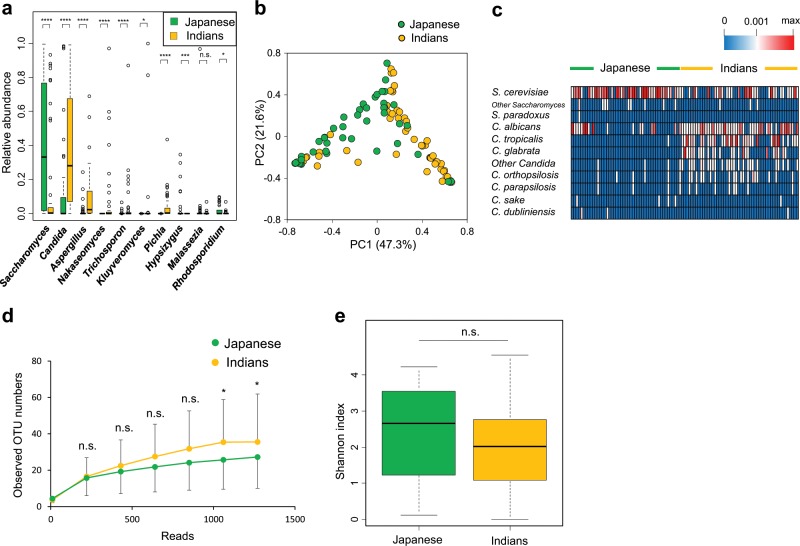
Comparison of fecal fungi in healthy adults living in Japan and India. **a** Relative abundances of the major genera. **b** Principal component analysis of fecal fungi at the genus level. **c** Heatmap representing the relative abundances of fungal species in genus *Saccharomyces* and *Candida*. **d** Rarefaction curves. **e** Shannon index. n.s. not significant; ****p < 0.0001; ***p < 0.001; *p < 0.05.

Next, we analyzed fecal fungi from individuals living in Japan and India using an improved sequencing procedure to analyze fungal composition by comparing the internal transcribed spacer 1 (ITS1) sequences of rRNA genes.^16^ Ascomycota and Basidiomycota were the main fungal phyla in both Japanese and Indian samples (Supplementary Fig. 1e, f). The relative abundance of Basidiomycota in Japanese samples was remarkably higher than that in Indians. *Saccharomyces* and *Candida*, both of which belong to the Ascomycota, were found to be the major fungal genera in Japanese and Indian samples, respectively (Fig. 2a). The PCA showed a clear separation between Japanese and Indian samples in terms of the variation of the fungal composition (Fig. 2b). For the genus *Candida*, the detection ratios and relative abundances of *C. albicans*, *Candida tropicalis*, and *Candida glabrata* in Indian samples were markedly higher than those in Japanese (Fig. 2c). Among *Saccharomyces*, *Saccharomyces cerevisiae* dominated in Japanese samples. Similar to bacterial case, the Shannon diversity indices for gut fungal species were similar in both Japanese and Indian subjects (Fig. 2d, e). Demographics such as age and sex were not associated with the bacterial and fungal composition (data not shown). Thus, the composition of both intestinal fungi and bacteria varied between individuals living in these distinct areas.

### Growth of Prevotella and Candida on several plant polysaccharides

**Fig. 3:**
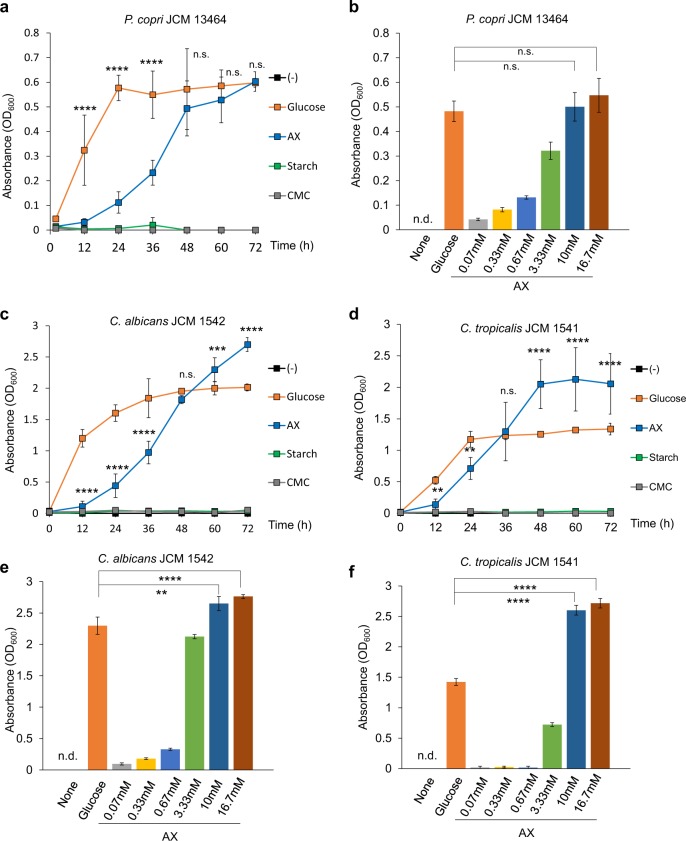
Co-utilization of arabinoxylan by *Candida* and *Prevotella*. **a**, **c**, **d** Growth of *P. copri* (**a**), *C. albicans* (**c**), and *C. tropicalis* (**d**) in the presence or absence of 10 mM glucose, arabinoxylan (AX), starch, or carboxymethyl cellulose (CMC). The growth rate in the presence of AX and glucose was statistically compared. **b**, **e**, **f** Growth response of *P. copri* (**b**), *C. albicans* (**e**), and *C. tropicalis* (**f**) in the presence or absence of 10 mM glucose or the indicated concentrations of AX at 72 h. (−) is base media alone without any carbon source. n.s. not significant, n.d. not detected. ****p < 0.0001; ***p < 0.001; **p < 0.01.

Given that dietary habits predominantly shape gut bacterial ecology,^9,23^ we reasoned that the components of plant-based diets might influence the composition of both the bacterial and fungal communities in the intestine. We analyzed the effect of plant polysaccharides (constituents of cereals), such as starch and dietary fibers, including wheat arabinoxylan (AX),^24^ and carboxymethyl cellulose (CMC), which is the soluble alternative to cellulose, on in vitro growth of *Prevotella* and *Candida*, both of which were dominant microorganisms in the intestines of Indian population we recruited. To monitor the growth yields of *P. copri* and *Candida* spp. (*C. albicans*, *C. tropicalis*, and *C. glabrata*), these microorganisms were cultured in a medium containing individual polysaccharides. Previous studies reported that glucose (a monosaccharide) supported the growth of these microorganisms when used as the sole carbohydrate source.^25–27^ Therefore, we used glucose as the sole carbohydrate in the growth medium and observed that *P. copri* grew rapidly in this condition (Fig. 3a, Supplementary Fig. 2a). In accordance with previous reports analyzing *Prevotella* spp. isolated from ruminant animals,^25,28,29^ *P. copri*, which was originally isolated from the human intestine^30^ and obtained from Japan Collection of Microorganisms (JCM), utilized AX as the sole carbon source and proliferated in the defined medium though at a slower rate than when using glucose. However, *P. copri* did not grow in response to other types of dietary polysaccharides tested in the current study. *P. copri* showed a dose-dependent growth response to AX and grew equally in the presence of AX or glucose (Fig. 3b). To confirm that the slower growth response of *P. copri* in the presence of AX is not strain-specific, we isolated *P. copri* from the feces of Indian subjects and analyzed the growth response (Supplementary Fig. 2b). The isolated *P. copri* showed the similar growth response to AX and glucose as the *P. copri* JCM strain, although it also responded to starch. Next, we analyzed the growth response of *Candida*, using publicly available type strains obtained from JCM, to the same sets of dietary polysaccharides, in yeast nitrogen base (YNB) medium. Similar to *P. copri*, both *C. albicans* and *C. tropicalis* grew in the presence of glucose or AX but not the other polysaccharides (Fig. 3c, d, Supplementary Fig. 3a). *C. albicans* and *C. tropicalis* achieved higher growth in the presence of AX than glucose at an equimolar concentration (10 mM) (Fig. 3c–f). We next isolated *Candida* species (*C. albicans*, *C. tropicalis*, and *C. glabrata*) from the fecal samples of Indian subjects. Although *C. glabrata* did not show a strong growth response to AX, *C. albicans* and *C. tropicalis* isolated from Indians showed the similar growth pattern as the JCM strains (Supplementary Fig. 3b–d), suggesting that the ability to utilize polysaccharides is preserved between the strains used in the current study. We also analyzed the growth responses of *S. cerevisiae* and *Bacteroides* species, such as *B. fragilis*, *B. ovatus*, *B. thetaiotaomicron*, and *B. uniformis*, which were dominant in Japanese samples (Supplementary Fig. 4a–e). *S. cerevisiae* did not show any growth response in the presence of the above mentioned dietary polysaccharides. *Bacteroides* species grew in the presence of starch, but not CMC, and some *Bacteroides* species showed a response to AX. Thus, intestinal microorganisms showed differential growth responses to various dietary polysaccharides.

### Candida-dependent dietary metabolites that support Prevotella growth

**Fig. 4:**
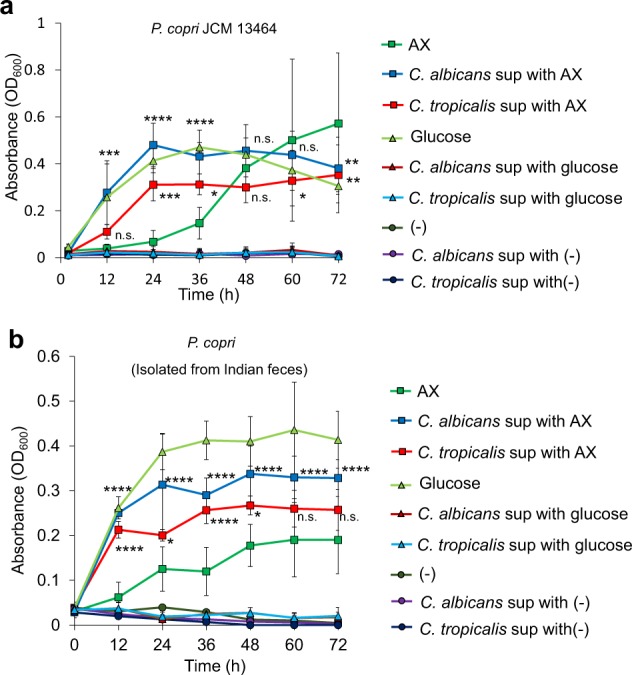
Promotion of *Prevotella* growth by the metabolites produced by *Candida.* Growth of *P. copri* JCM 13464 (**a**) and isolates from Indian feces (**b**) in the presence of glucose, AX, and *C. tropicalis*- or *C. albicans*-supernatants from cultures grown in AX. (−) is base media alone without any carbon source. Results of statistical comparison between *C. albicans* and *C. tropicalis*-supernatants from cultures grown in AX with AX alone are shown. All the graphs show the mean ± SD of three independent experiments. n.s. not significant. ****p < 0.0001, ***p < 0.001, **p < 0.01, *p *<* 0.05.

We next analyzed the interaction of *Candida* and *Prevotella*, both of which dominated the intestine of Indian subjects and showed growth response to AX. Given the effective use of AX by *Candida* over *Prevotella*, we analyzed whether *Candida* supports *Prevotella* growth in an AX-rich environment (Fig. 4a). Addition of culture supernatants of *C. albicans* or *C. tropicalis* grown in the presence of AX induced rapid growth of *P. copri* compared to its growth in the presence of AX alone. Similarly, *Candida* strains isolated from Indian feces promoted *P. copri* growth (Fig. 4b). These results suggest that the fungal supernatants were enriched in metabolic products that enabled rapid growth of *P. copri*.

**Fig. 5:**
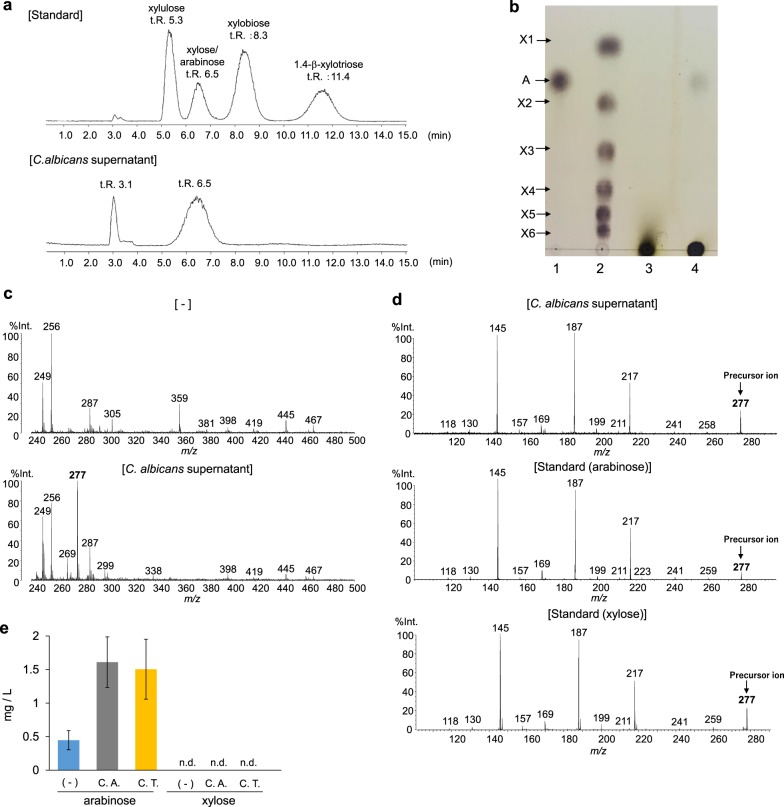
Identification of arabinose generated from *Candida*-dependent arabinoxylan metabolism. **a** HPLC chromatograms of standards (upper: xylulose, xylose, xylobiose, xylotriose, and arabinose) and the *C. albicans* culture supernatant (lower). **b** TLC analyses of the *C. albicans* culture supernatant. Lane 1: arabinose (A); Lane 2: xylose (X1), xylobiose (X2), xylotriose (X3), xylotetraose (X4), xylopentaose (X5) and xylohexaose (X6); Lane 3: yeast nitrogen base medium with AX; Lane 4: culture supernatant of *C. albicans* grown in the presence of AX. **c** Direct mass spectrometry analysis of the spot detected in TLC analysis (Lane 4). Mass spectra of negative control (−) and sample spot of the TLC plates used for *C. albicans* culture supernatant. A unique ion peak was observed at *m/z* 277 in the *C. albicans* supernatant sample. **d** MS/MS spectra of the precursor ion at *m/z* 277 in the *C. albicans* supernatant sample (upper), and for arabinose (middle) and xylose (lower). The MS/MS fragment patterns of the *C. albicans* supernatant sample were identical to those of xylose and arabinose. **e** Concentration of D-xylose and l-arabinose in the medium with AX (−) and *Candida* culture supernatants with AX. C.T., *C. tropicalis*; C.A, *C. albicans*. Data are shown as means ± SD from three independent experiments. n.d. not detected.

**Fig. 6:**
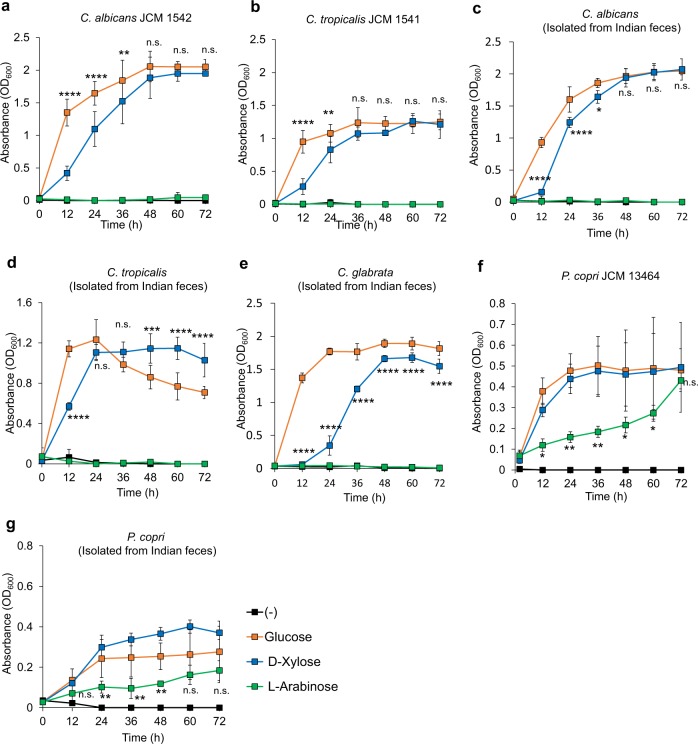
In vitro growth of *Candida* and *Prevotella* in response to monosaccharides. **a**–**g** Growth of *C. albicans* JCM 1542 (**a**), *C. tropicalis* JCM 1541 (**b**), *C. albicans* isolated from Indian feces (**c**), *C. tropicalis* isolated from Indian feces (**d**), *C. glabrata* isolated from Indian feces (**e**), *P. copri* JCM 13464 (**f**), and *P. copri* isolated from Indian feces (**g**) in the presence of 10 mM glucose, d-xylose or l-arabinose. Data from three independent experiments are shown as means ± SD. Statistical comparison between glucose and d-xylose (**a**–**e**) and between glucose and l-arabinose (**f**, **g**) is shown. n.s. not significant. ****p < 0.0001, **p < 0.01, *p < 0.05.

Subsequently, we attempted to identify the molecule in the culture supernatants of *Candida* grown in AX-containing medium that stimulated *P. copri* growth. First, the *C. albicans* culture supernatant was analyzed by high performance liquid chromatography (HPLC) (Fig. 5a). Because AX is a polymer of β-1,4 linked d-xylopyranosyl residues that are substituted with monomeric α-l-arabinofuranose units at the second and/or third carbon (C-2 and C-3) positions,^31,32^ xylo-oligosaccharides or monomeric d-xylose and l-arabinose were expected to be produced by AX degradation. Therefore, they were used as standards. In the *C. albicans* supernatant, two peaks were observed in HPLC with retention times of 3.1 and 6.5 min; the retention time of 6.5 min corresponded to that of xylose and arabinose. In an effort to distinguish between xylose and arabinose, supernatants of *C. albicans* (the JCM strain and the Indian fecal isolate) were separated by thin layer chromatography (TLC) (Fig. 5b, Supplementary Fig. 5a). A spot of the *C. albicans* supernatant was observed at a similar position to that of the arabinose standard, while no spots were observed comigrating with the xylose standard. A similar peak at retention time of 6.5 min in HPLC and spot in TLC were observed in supernatants of *C. tropicalis* (the JCM strain and the Indian isolate) (Supplementary Fig. 5b–d). Next, the TLC spot of the *C. albicans* supernatant was isolated and analyzed by mass spectrometry (MS) (Fig. 5c, d). A specific and strong signal was observed at *m/z* 277 in the *C. albicans* supernatant, and MS/MS spectra of the *m/z* 277 ion showed the same pattern as that of xylose and arabinose. These data collectively suggest that the *C. albicans* supernatant contained arabinose. We then measured the concentrations of d-xylose and l-arabinose in the *Candida* culture supernatants using xylose- or arabinose/galactose-specific enzyme-based colorimetric assays, respectively (Fig. 5e). Xylose was not detected in the supernatants of *C. albicans* or *C. tropicalis* cultures. In contrast, arabinose was elevated when *C. albicans* or *C. tropicalis* were cultured in the presence of AX. These findings indicate that arabinose was enriched in the *Candida* spp. supernatant, although AX degradation is expected to produce both xylose and arabinose. Therefore, we speculated that xylose produced by hydrolysis of AX was rapidly and completely consumed by *Candida* spp. To assess this, we analyzed the growth response of *Candida* (the JCM strain and the Indian isolate) to the monosaccharides d-xylose and l-arabinose (Fig. 6a–e). d-xylose, but not l-arabinose, induced prominent growth of both *C. albicans*, *C. tropicalis*, and *C. glabrata* at a level similar to that induced by glucose. These findings indicate that *Candida* strains used in the current study metabolize AX and use xylose for their growth. Then, we analyzed the effect of l-arabinose on the growth of *P. copri* (the JCM strain and the Indian isolate) (Fig. 6f, g). *P. copri* showed a marked growth response to l-arabinose as well as d-xylose. Consumption of arabinose by *P. copri* was also assessed by TLC (Supplementary Fig. 6). The spot corresponding to arabinose detected in the supernatant of *C. tropicalis* culture in the presence of AX was not detected in the culture supernatant of *P. copri* grown in the *C. tropicalis* AX supernatants. Thus, the AX-derived metabolite, which was produced by *Candida* and induced substantial growth of *P. copri*, could be arabinose. However, we should consider that several other microbes participate in utilizing such diet-derived metabolic products in the gut. Indeed, similar to *P. copri*, *Bacteroides* species also utilized l-arabinose (Supplementary Fig. 7), which indicate the presence of an unknown mechanism that might facilitate the preferential selection of *P. copri* by *Candida* (see Discussion).

### Promotion of Prevotella colonization by Candida in germ-free mice

**Fig. 7:**
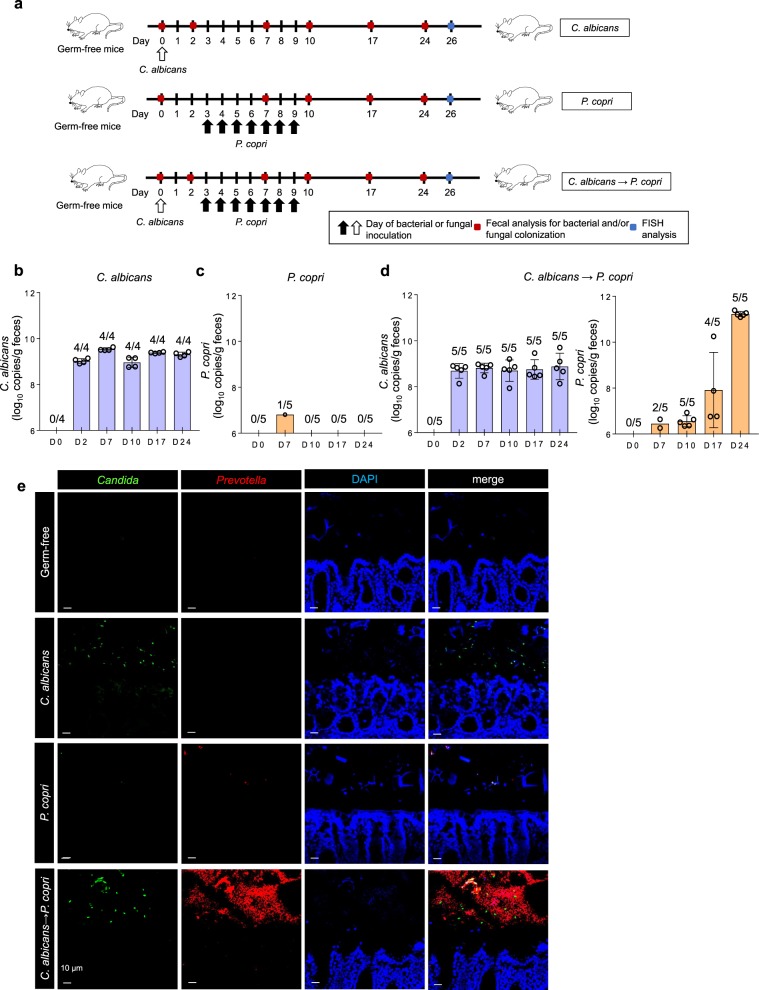
In vivo interaction of *Prevotella* and *Candida* in colonization of the mouse intestine. **a** Schematic diagram of fungal and bacterial administration in the mouse intestine: germ-free BALB/c mice were administered with *C. albicans* (*n* = 4), *P. copri* (*n* = 5) or *C. albicans* + *P. copri* (*n* = 5). *C. albicans* was orally administered on day 0, and *P. copri* was orally administered on days 3–9. **b–d** Copy numbers of *C. albicans* (**b**, **d**) and *P. copri* (**c**, **d**) per gram of feces at the indicated time points (days) in mono- and co-administered groups. The number of mice, in which the copy numbers of the microorganisms were above the detection limit, is indicated on the graph. Data are representative of two independent experiments and are shown as means ± SD. The mean values are calculated based on copy numbers that were above the detection limit. **e** FISH using *Candida*-specific probe Dual 1249 (green), *Prevotella*-specific probe PRV392 (red), and 4′, 6-diamidino-2-phenylindole (DAPI; blue) on Carnoy’s fixed colon sections harvested from mice 26 days after the initial colonization. Scale bars, 10 µm.

Next, we analyzed the in vivo interaction of *Candida* and *Prevotella* using a gnotobiotic mouse colonization model. Germ-free (GF) mice were orally administered *Candida* or *Prevotella* and then the microbial load in feces was analyzed (Fig. 7a). *C. albicans* and *C. tropicalis* successfully colonized the mouse intestine, reached a peak level within 2 days post administration and maintained peak numbers for >3 weeks (Fig. 7b, Supplementary Fig. 8). In contrast, *P. copri* was barely detectable, indicating that *Prevotella* had limited ability to colonize the mouse intestine on its own (Fig. 7c). In the next set of experiments, GF mice were first colonized with *C. albicans* and then *P. copri* was orally administered 3 days later. In the *Candida*-enriched intestinal environment, *P. copri* increased gradually and outnumbered *Candida* at day 24 (3 weeks after the *P. copri* administration) (Fig. 7d). We also analyzed the localization of both microorganisms in the colon by fluorescence in situ hybridization (FISH) using probes specific for *Prevotella* and *Candida* (Fig. 7e). In mice administered *C. albicans* alone, *Candida* was detected in the colonic lumen. In contrast, *Prevotella* was barely detected in the colonic lumen of mice given *P. copri* alone. However, elevated numbers of *Prevotella* were observed in the colonic lumen and feces of mice administered both *C. albicans* and *P. copri* (Fig. 7e, Supplementary Fig. 9). These findings indicate that *P. copri* efficiently colonized and grew in the intestine in the presence of *Candida* spp.

## Discussion

Our study analyzed the intestinal bacterial and fungal composition of Japanese and Indian adults and provided evidence for an interkingdom interaction that is potentially mediated by differences in the host diet. Japanese populations, with high intake of animal products, showed an abundance of *Bacteroides* compared to Indians, consuming plant-based diets, showing higher levels of *Prevotella*. These observations are parallel to the earlier findings in human populations with a diet enriched in complex carbohydrates, such as Hadza hunter-gatherer from Tanzania^33^ and children from rural Africa^6^ who showed a higher abundance of *Prevotella* compared to populations ingesting a western diet harboring higher levels of *Bacteroides*. In accordance with those studies, *Prevotella* has been shown abundant in individuals with high intake of carbohydrates/dietary fiber.^23^

In addition to bacteria, accumulating evidence indicates that other domains of life residing in the gut, including viruses,^34,35^ archaea^36^ and eukaryotes, such as protozoans^37^ and fungi,^36,38^ contribute to the development of gut ecosystem and influence the host physiology. Indeed, the correlation of dietary habits with the composition of intestinal archaea and fungi has been reported.^36^ However, further studies are required to establish the mechanisms of their interaction with one another in the complex gut environment. A previous study in mice showed that commensal bacteria *B. thetaiotamicron* and *Blautia producta* can promote colonization resistance to *C. albicans* by increasing expression of antimicrobial peptide LL-37 mediated by hypoxia-inducible factor-1α.^39^ Another study showed that human gut *Bacteroides* has the glycoside phosphorylase genes that targets β-1,2-mannnosidic linkages in *Candida* mannan, and it can utilize yeast mannan as a food source.^40^ It was also reported that oral administration of *Saccharomyces* in obese mice resulted in alteration of bacterial composition, in which *Bacteroides* was dramatically increased and *Prevotella* was decreased.^41^ These findings could potentially explain the observation in our study where Japanese cohort had a substantial presence of *Saccharomyces*, *Bacteroides*, and *Blautia* but lower levels of *C. albicans*, and Indian cohort had lower levels of *Bacteroides*. Thus, it will be crucial to carefully study the host-derived influences together with external factors including diet in order to completely assess the differential colonization of microbial communities across populations.

The information from dietary habitat survey indicated that diets of Indian subjects were rich in plant-derived carbohydrates (fiber-rich) and therefore representative dietary plant polysaccharides were included for in vitro growth response assay of *Prevotella* and *Candida*. Xylan, second most abundant (after cellulose) plant polysaccharide, is a known substrate for microbial fermentation in the gut of ruminants as well as humans.^42^ Cereal grains, such as wheat, corn, and rye, have higher proportion of xylan.^43^ *P. copri*, like ruminant origin *P. bryantii*,^25^ grew in response to wheat AX. This is the first study that tested the growth response of *Candida* spp. (both JCM and Indian fecal isolates) toward AX. *Candida* utilized AX from the panel of three polysaccharides and generated arabinose which potentially enhanced *P. copri* growth in vitro, suggesting a dietary metabolite mediated interaction between fungi and bacteria. It is important to note that while we observed that arabinose was produced by *Candida* and consumed by *P. copri*, the in vitro assays tested for specific metabolites. Thus, a comprehensive characterization of the metabolites is warranted to gain deeper insights into the dietary components mediating microbial interactions.

Similar to in vitro observations, this interkingdom interaction was recapitulated in GF mouse system where we observed increased *P. copri* numbers in the presence of *Candida*. However, given the multitude of factors regulating highly dynamic and complex intestinal system, other mechanisms might be active to facilitate this interaction. For instance, some intestinal bacteria, such as *Bacteroides*, have been known to ferment polysaccharides of the yeast cell wall such as mannan^40^ and β-glucans.^44^ Thus, it is also possible that in addition to diet-derived sources *Prevotella* might benefit from the presence of *Candida* by other alternate mechanisms. In the present study, we focused on validating the interkingdom interaction and proposed arabinose as a potential candidate. However, additional experiments will be required to establish its role as a major beneficiary module that facilitates bacterial growth. For instance, future studies comparing GF mice colonized with respective microbes by administering a customized diet rich in AX and AX-free diet group will be able to provide a clearer picture of the impact of AX/arabinose based cross-feeding mechanism in colonization of *Prevotella* in the intestine.

AX itself has a complex chemical structure comprised of linear d-xylose backbone. In various grain species, the backbone xylose may be substituted by arabinose and cross-linked with ferulic acid. To acquire energy, microbes need to depolymerize the polysaccharides by enzymatic cleavage of the chemical linkages. Gut bacteria produce thousands of substrate-specific carbohydrate-active enzymes (CAZymes) that catalyze the breakdown of the unique linkages and have been extensively cataloged.^28,45,46^ Thus, one of future directions would be identification of CAZymes in *Candida* spp. and *Prevotella* for utilizing AX.

The present study aims to highlight the importance of analyses on the lesser-studied microorganisms, particularly fungi, for a comprehensive understanding of the complex interactions in the gut microbial ecosystem. Although our study mainly focused on *Candida* species, which identified them as AX-degraders, there are several AX-degrading microorganisms including *Bacteroides* species, *Eubacterium rectale*, and *Bifidobacterium* species in the human intestine.^42,47–49^ Considering a physiological intestinal environment, in which these bacterial taxa outnumber *Candida* spp., AX degradation in the gut ecosystem is likely to be a much more complicated process. It will, therefore, be necessary in the future to design a gnotobiotic system with curated complex microbial communities for dissection of the cross-feeding behavior. Our study presented one of the plausible mechanisms by which a fungus might facilitate the growth of a bacterium in the intestine. Thus, an analysis of complex association of different microorganisms is better explored further in the future by taking account of the fact that the gut contains complex microbial consortia consisting of several domains of life. In this study, the colonization of *Candida* is shown to help *Prevotella* growth potentially through AX metabolites. It will be interesting to study whether the interaction of these microorganisms in the intestine is involved in the maintenance of the host health.

## Materials and methods

### Fecal collection and processing

Fecal samples were collected from 47 healthy Japanese adults living in the Osaka area (25 males and 22 females, average age 30.6 ± 6.1 years) and 50 healthy Indians living in the Delhi area (27 males and 23 females, average age 28.8 ± 6.2 years). A spoonful of feces (0.5 g) was collected into a tube containing 2 ml of RNA*later* (Ambion) for nucleic acid extraction. Collections were made immediately after defecation. Each fecal sample for nucleic acid extraction was weighed and suspended in nine volumes of RNA*later* to make a fecal homogenate (100 mg feces/ml). In accordance with the Declaration of Helsinki, all subjects were adequately informed about the study. Informed written consent was collected from all the participants. The ethics committees of Osaka University and the Translational Health Science and Technology Institute (Faridabad) approved this study. The protocol numbers are 12237, and SAS/THSTI/001/2013-2014, respectively. The samples were transported between Japan and India in accordance with the Nagoya protocol.

### Extraction of DNA for bacterial analysis

For DNA extraction, 1 ml of phosphate-buffered saline (PBS) was added to 200 μl of fecal homogenate. The fecal homogenate was centrifuged at 13,000 × *g* for 10 min and 1 ml of the supernatant was discarded. After another wash with 1 ml of PBS, the pellets were stored at −30 °C until use for DNA extraction. Glass beads (0.3 g; diameter, 0.1 mm) (BioSpec Products), 300 μl Tris-SDS solution and 500 μl Tris-EDTA (TE)-saturated phenol were added to 200 μl of the fecal homogenate, and the mixture was vortexed vigorously for 30 s using a FastPrep-24 (M.P. Biomedicals) at 5.0 power level for 30 s. After centrifugation at 20,000 × *g* for 5 min at 4 °C, 400 μl of the supernatant was collected and an equal volume of phenol-chloroform-isoamyl alcohol (25:24:1) was added to the supernatant. After centrifugation at 20,000 × *g* for 5 min at 4 °C, 250 μl of the supernatant was collected and subjected to isopropanol precipitation. Finally, the DNA was suspended in 200 μl of TE buffer and stored at −30 °C.

### Determination of bacterial composition by MiSeq amplicon sequencing

Each DNA library was prepared according to the “Illumina 16S Metagenomic Sequencing Library Preparation Guide” with primer set 27Fmod: 5ʹAGRGTTTGATCMTGGCTCAG-3ʹ and 338R: 5ʹ-TGCTGCCTCCCGTAGGAGT-3ʹ targeting the V1–V2 region of 16S rRNA genes; 251-bp paired end sequencing of the amplicons was performed on a MiSeq system (Illumina) using a MiSeq Reagent v2 500 cycle kit. The paired end sequences obtained were merged using PEAR (http://sco.h-its.org/exelixis/web/software/pear/). Subsequently, 30,000 reads per sample were randomly sampled according to the minimum read in a sample using seqtk (https://github.com/lh3/seqtk) for taxonomic assignment. These sampled sequences were then clustered into OTUs defined at 97% similarity cutoff using UCLUST version 1.2.22q. Representative sequences for each OTU were classified taxonomically using RDP Classifier version 2.2^50^ with the Greengenes database (gg_13_8). The Mann–Whitney *U* test was conducted for statistical analyses by using R 3.2.2. Although the rarefaction curves did not reach saturation (Fig. 1d) due to the limited reads in a sample, we could observe the tendency that the observed OTU numbers in Indians were higher than those in Japanese at all the points.

### Extraction of DNA for fungal analysis

Five-hundred microliters of fecal homogenate (50 mg feces) were washed twice with 1 ml of PBS and fungal DNA was extracted by using the PowerSoil DNA isolation kit (MO BIO Laboratories) according to the manufacturer’s protocol. The fungal DNA was stored at −20 °C until use. Polymerase chain reaction (PCR) was performed with primers ITS1-F (5′-CTTGGTCATTTAGAGGAAGTAA-3′) and ITS2 (5′-GCTGCGTTCTTCATCGATGC-3′), which are specific to the fungal ITS1 region.^16^ Each reaction mixture (50 μl) was composed of 1× PCR buffer, each deoxynucleoside triphosphate at 200 μM, each primer at 0.4 μM, 2.5 units of rTaq (Takara), and 1 μl of fungal DNA as the template. The amplification program consisted of one cycle at 95 °C for 2 min, 40 cycles at 95 °C for 20 s, 56 °C for 30 s, and 72 °C for 30 s, followed by 1 cycle at 72 °C for 10 min. The PCR products containing the fungal ITS1 region, whose length was widely distributed from approximately 250–700 bps, were purified and subjected to Single Molecule Real-Time (SMRT) sequencing using a PacBio RSII instrument (Pacific Biosciences).

### Determination of fungal composition by PacBio technology

A DNA library was prepared using the DNA Template Prep kit 2.0 (Pacific Biosciences) according to the manufacturer's instructions. Sequencing was performed with the PacBio RS II system using the DNA Sequencing Kit C2 (Pacific Biosciences) with P4 polymerase. Circular Consensus Sequence (CCS) constructed from more than eight full-pass subreads were produced using PacBio SMRT Analysis, and then primer sequences were removed using the FASTX-Toolkit (http://bbmap.sourceforge.net/). For fungal analyses, 2202 reads in average in 1 sample were generated. Sequences were clustered into OTUs, defined at 95% similarity using UCLUST version 1.2.22q (http://sco.h-its.org/exelixis/web/software/pear/). Representative sequences for each OTU were classified taxonomically using RDP Classifier version 2.2 with the ntF-ITS1 database.^16^ The Mann–Whitney *U* test and the Fisher’s probability test were used to compare the relative abundance and detection ratio for statistical analyses, respectively.

### Microorganisms

Bacteroidales strains used in this study, *P. copri* JCM 13464^T^, *B. fragilis* JCM 11019^T^, *B. ovatus* JCM 5824^T^, *B. thetaiotaomicron* JCM 5827^T^, and *B. uniformis* JCM 5828^T^ were obtained from the Japan Collection of Microorganisms (JCM). The fungal strains *C. tropicalis* JCM 1541^T^, *C. albicans* JCM 1542^T^, and *S. cerevisiae* JCM 7255^T^ were also obtained from JCM. For isolation of *P. copri* strains from Indian feces, fecal dilutions with PBS were spread on sheep blood agar (BD) and cultured in an anaerobic condition at 37 °C for 48 h. The colonies were picked up and subjected to colony PCR using g-Prevo-F (5′-CACRGTAAACGATGGATGCCCACRGTAAACGATGGATGCC-3′) and g-Prevo-R (5′-GGTCGGGTTGCAGACC-3′).^51^ The positive colonies were again subjected to colony PCR using 8F (5′-AGAGTTTGATCMTGGCTCAG-3′) and 15R (5′-AAGGAGGTGATCCARCCGCA-3′)^52^ targeting full length of 16S rRNA gene. After purification of the amplicon by using GEL/PCR Purification Mini Kit (FAVORGEN), a full length of 16S rRNA gene were analyzed using BigDye Terminator (Applied Biosystems) on ABI 3730 sequencer (Applied Biosystems) by the following primer: 520R (5′-ACCGCGGCTGCTGGC-3′), 520F (5′-CAGGAGTGCCAGCAGCCGCGG-3′), 800R (5′-CAGGACTACCAGGGTATCTAAT-3′), 930F (5′-GCACAAGCGGTGGAGCATGTGG-3′), or 1100R (5′-AGGGTTGCGCTCGTTG-3′).^52^ For isolation of *Candida* strains, fecal dilutions with PBS were spread on potato dextrose agar (Merck) with 0.05% (w/v) chloramphenicol and cultured in an aerobic condition at 37 °C for 48 h. The colonies were picked up and subjected to colony PCR using the following primer: UNI1 (5′-ATGAAGAACGCAGCGAAATGCGATA-3′) and UNI2 (5′-GTTGGTTTCTTTTCCTCC-3′).^53^ The PCR product was purified by GEL/PCR Purification Mini Kit (FAVORGEN) according to the manufacture’s protocol. The ITS2 region was sequenced by using the BigDye Terminator (Applied Biosystems) with the FSeq (5′-ATGCCTGTTTGAGCGTC-3′) or RSeq (5′-CCTACCTGATTTGAGGTC-3′)^53^ on ABI 3730 sequencer (Applied Biosystems). Three strains of *P. copri*, and 2, 5, and 10 strains of *C. albicans*, *C. tropicalis*, and *C. glabrata* were successfully isolated from Indian fecal samples, respectively. One representative strain from *Prevotella* and *Candida* isolates was used for experiments.

### Reagents

Wheat AX (medium viscosity, 31 centistokes) was obtained from Megazyme, CMC from Nacalai Tesque and soluble starch from Sigma-Aldrich. Monosaccharides d-xylose and d-glucose were purchased from Nacalai Tesque. Xylulose and l-arabinose were purchased from Sigma-Aldrich. Xylo-oligosaccharides (xylobiose, xylotriose, xylotetraose, xylopentaose, and xylohexaose; X1–X6) as standards for TLC were purchased from Megazyme and Silica Gel 60 F254 TLC plates (5 cm × 10 cm) were obtained from Merck. Colorimetric detection reagent, orcinol monohydrate, was purchased from Sigma-Aldrich. α-Cyano-4-hydroxycinnamic acid (CHCA) and 2,5-dihydroxybenzoic acid (DHB) were purchased from Shimadzu GLC. Angiotensin II, 3-aminoquinoline (3-AQ), and N-acetyl-renin substrate were purchased from Sigma-Aldrich. Ammonium dihydrogen phosphate was purchased from Merck Millipore. Trifluoroacetic acid (TFA) was purchased from Wako Pure Chemical Industries.

### In vitro growth of bacteria and fungi

For experiments analyzing growth response toward polysaccharides and monosaccharides, bacterial strains were grown in a modified chemically defined medium as described previously^25^ with the addition of tryptone (0.2%; BD Biosciences). First, *P. copri*, *B. fragilis*, *B. ovatus*, *B. thetaiotaomicron*, and *B. uniformis* were cultured on blood agar plates from their glycerol stocks. Single colonies of the bacteria were picked individually and cultured in a Ruskinn Bugbox Plus anaerobic chamber (The Baker Company; 10% H_2_, 10% CO_2_, 80% N_2_) at 37 °C overnight in the 4 ml Gifu Anaerobic Medium (GAM) (OD_600_ 1.0–1.5). The bacterial cultures were then pelleted by centrifugation at 1300 × *g* for 5 min. Culture pellets were washed and re-suspended in 1× modified chemically defined medium. Twenty microliters of the culture were then added to 2 ml modified chemically defined medium supplemented with either AX or starch or CMC or d-glucose or d-xylose or l-arabinose as the sole carbohydrate source as indicated. The final concentration of all the carbohydrates used corresponded to 10 mM, as reported in the growth studies of ruminant *Prevotella bryantii*.^25^ For dose dependent growth analysis towards AX, 0.07–16.7 mM monosaccharide equivalents were used as the sole carbohydrate source. To assess the growth response of fungi in the presence of dietary polysaccharides and monosaccharides, YNB medium (BD Difco) was used. Initially, *C. tropicalis*, *C. albicans*, *C. glabrata*, or *S. cerevisiae* were cultured in yeast extract, peptone and dextrose (YPD) broth (BD Biosciences) in an aerobic environment at 30 °C. Subsequently, the cultures were pelleted and washed using 1× YNB medium. Similar to bacteria, 20 μl of fungal cultures grown overnight were inoculated into 2 ml of YNB supplemented with the above mentioned carbohydrate. For experiments assessing the interaction between *Candida* and *Prevotella*, the 72 h culture supernatants of *C. tropicalis* or *C. albicans* grown in the presence of glucose or AX were filter sterilized (0.22 μm) and added to the modified chemically defined medium in the ratio of 3:1 (v/v). Growth rates were assessed by measuring optical density at 600 nm wavelength using a biophotometer (Eppendorf) over a period of 72 h with readings taken at multiple time points. All the experiments were performed in triplicate. For statistical analysis, two-way analysis of variance with Dunnett’s post hoc test was performed using GraphPad Prism (version 7.01 for Windows, GraphPad Software, La Jolla, CA, USA).

### Measurement of arabinose and xylose concentration

The 72 h culture supernatants of *C. albicans* or *C. tropicalis* (5 ml) were concentrated by evaporating to dryness using an EZ-2 Plus Genevac centrifugal evaporator (SP Scientific) and the dried contents were dissolved in 250 µl of distilled water (20-fold concentrated). The concentrations of liberated arabinose and xylose were quantified using the l-arabinose/d-galactose assay kit (K-ARGA, Megazyme) and the d-xylose assay kit (K-XYLOSE, Megazyme), respectively.

### High performance liquid chromatography

*C. albicans* and *C. tropicalis* were grown in the presence of AX for 72 h. The culture supernatants (5 ml) were then evaporated using an EZ-2 Plus Genevac centrifugal evaporator. The dried samples were dispersed in water and then filtered to remove insoluble solids before HPLC analysis. HPLC was performed using a Shimadzu Prominence HPLC system equipped with a Softa 400 ELSD detector and a COSMOSIL Sugar-D column (Φ4.6 mm × 250 mm; mobile phase: CH_3_CN/H_2_O(3/1), flow rate: 1.0 ml/min, temperature: 30 °C).

### Thin layer chromatography

The capacity of *C. albicans, C. tropicalis*, and *P. copri* to hydrolyze AX or AX-derived metabolites was assessed by resolving and detecting the hydrolysis products using TLC. *C. albicans* and *C. tropicalis* were grown in the presence of AX for 72 h. *P. copri* was grown in the culture supernatant of *C. tropicalis* in the presence of AX. The culture supernatants (5 ml) were evaporated using an EZ-2 Plus Genevac centrifugal evaporator. The dry matter was then resuspended in 100 µl of distilled water and 2 µl was spotted onto a DC-Kieselgel Silica Gel 60 F254 TLC plate to resolve the products. Monomeric xylose (X1), and xylo-oligosaccharides (X2–X6) (0.5 mg/ml each) and arabinose (1.5 mg/ml) were used as standards. TLC plates were developed using an *n*-butanol: acetic acid: distilled water (10:5:1 v/v/v) as an eluent.^54,55^ The products were then visualized by spraying the plates with a 1:1 (v/v) mixture of methanolic orcinol (0.2% w/v) and sulfuric acid (20% v/v) followed by heating the plates at 100 °C for 5 min.^28,56^

### Mass spectrometry

Mass spectrometry analysis was performed with a matrix-assisted laser/desorption ionization quadrupole ion trap time-of-flight (MALDI-QIT-TOF) mass spectrometer (AXIMA Resonance; Shimadzu/Kratos) in the positive-ion mode. Ionization was performed with a 337 nm pulsed N_2_ laser. Helium and argon gases were used for ion cooling and collision-induced dissociation, respectively. A matrix solution was prepared by dissolving 10 mg DHB in 1 ml of 50% acetonitrile containing 0.1% TFA aqueous solution. As calibrants for the instrument, angiotensin II and N-acetyl-renin substrate were dissolved in 30% acetonitrile containing 0.1% TFA aqueous solution to 10 pmol/μl. The two peptide solutions were mixed, and 0.5 μl of the mixed solution and 0.5 μl of DHB matrix solution were deposited onto a MALDI target plate sequentially. A liquid matrix 3-AQ/CHCA was prepared by mixing 3-AQ and CHCA based on a procedure as described previously.^57^ Briefly, CHCA solution was prepared by dissolving 10 mg of CHCA in 600 μl of 50% acetonitrile containing 10 mM ammonium dihydrogen phosphate solution, then 20 mg of 3-AQ was dissolved in 150 μl of CHCA solution, and diluted 10-fold using 50% acetonitrile aqueous solution. *C. albicans* culture supernatant (15 ml, after culture for 72 h in AX-containing medium) was evaporated and resuspended in 500 µl of distilled water; 1 µl was spotted onto a TLC plate to resolve the products. The surfaces of the sample spot areas (indicated as *C. albicans* in Fig. 5c) on five TLC plates were scraped off and collected into a polypropylene microtube, and the surface of a TLC plate without sample loading (indicated as (−) in Fig. 5c) was collected into another microtube for use as the negative control. The collected silica was suspended in 1 ml of water, shaken at room temperature for 20 min, and centrifuged at 20,000 × *g* for 10 min. The supernatant was collected into a new microtube, concentrated to dryness using a centrifugal concentrator (SPD-2010; Thermo Scientific), and reconstituted in 10 μl of water. An aliquot of 0.5 μl of the analyte solution was mixed with an equal volume of 3-AQ/CHCA matrix solution. The mixed solution was deposited onto the MALDI target plate and incubated at 60 °C for 1 h on a heating block (ALB-121; Scinics). By means of this preparation step, saccharides were labeled with 3-AQ. After the target plate was cooled to room temperature, it was introduced into the instrument and the analyte was measured. The instrument was calibrated using H adducted ions of angiotensin II ([M + H]^+^, *m*/*z* = 1046.54) and N-acetyl-renin substrate ([M + H]^+^, *m*/*z* = 1800.94) before sample analysis.

### Mice

GF (IQI/Jic[Gf] ICR as well as BALB/c) mice were purchased from CLEA, Japan. All mice were maintained in GF conditions at the Experimental Animal Facility, Graduate School of Medicine, Osaka University. All animal experiments were performed in accordance with the guidelines of the Animal Research Committee at Osaka University. The protocol number is DOUI28-026-007.

### Colonization of *P. copri* and *C. albicans* in GF mice

To assess the interaction between *P. copri* and *C. albicans* in mice, we prepared three separate groups of GF mice which was based on the administration of *P. copri* JCM 13464^T^ and *C. albicans* JCM 1542^T^ as indicated in the schematics of Fig. 4a. In the first group, GF mice were gavaged with *C. albicans* suspension (10^9^ colony forming units [CFU]/mouse) alone. This day of *C. albicans* gavaging was labeled day 0 to indicate the starting point of the experiment. In the second group, the mice were orally administered with *P. copri* suspension (about 10^10^ CFU of bacteria/mouse) alone. *P. copri* administration started on day 3 and continued for 7 consecutive days (i.e., until day 9).^58^ Finally, the mice in the third group were gavaged with *C. albicans* on day 0 using the same suspension as the first group. Similar to the second group, *P. copri* was administered from day 3 until day 9, using the same *P. copri* suspension that was used for the second group. All the three experimental groups were kept separate and provided with CRF-1 diet, which contains 3.1 g/100 g of fiber derived from wheat and alfalfa (Oriental Yeast Co., Ltd.). The experiment was performed twice independently using Jcl-ICR GF male mice (10–13 weeks old, 3 mice per group) or BALB/c GF male mice (13–17 weeks old, 4 or 5 mice per group). For preparing an oral suspension, *C. albicans* was cultured in 7.5 ml YPD medium for 16 h and centrifuged at 1870 × *g* for 5 min. The pellet was resuspended in 10 ml PBS and transferred to the mouse facility in 1.5 ml screw cap tubes. The *C. albicans* suspension of 200 μl was orally administered to mice in both the first and third groups on day 0. Next, *P. copri* suspension was prepared by culturing single colony of *P. copri* (obtained on a blood agar plate from the glycerol stock) for 12 h in 7.5 ml GAM broth. The culture was centrifuged at 1870 × *g* for 5 min and then resuspended in 2.5 ml prereduced 1× PBS. Tightly sealed 1.5-ml tubes containing the *P. copri* suspension were transported to the mouse facility in an AnaeroPack Rectangular Jar (Mitsubishi Gas Chemical Company, Inc.) to ensure anaerobic conditions during transportation. For both the second and third groups 200 μl of *P. copri* suspension was orally administered per mouse, from day 3 until day 9, using the same culture procedure. Fecal samples were collected at the indicated time points (Fig. 7a) and DNA was extracted as described in bacterial DNA extraction section above, except bead size used for fungal DNA extraction was 1.0 mm.

### Quantitative PCR

For enumeration of *P. copri, C. albicans*, and *C. tropicalis* in mouse samples by quantitative PCR (qPCR), the following primers sets were used: PCFw (5′-CCGGACTCCTGCCCCTGCAA-3′) and PCRv (5′-GTTGCGCCAGGCACTGCGAT-3′) for *P. copri*.^59^ Candida_albicans_138Fw (5′-GCCGCCAGAGGTCTAAACTT-3′) and Candida_albicans_234Rv (5′-GAACCAAGAGATCCGTTGTTGA-3′) for *C. albicans*^16^; and Ctro (5′-TATTGAACAAATTTCTTTGGTGGC-3′) and UNI2 (5′-GTTGGTTTCTTTTCCTCC-3′) for *C. tropicalis*.^53^ qPCR assays were performed in 96-well optical plates (Watson Biolab). Each reaction consisted of 5 μl of 10-fold diluted DNA as the template and 15 μl of master mix solution (4.6 μl PCR-grade water, 0.2 μl forward primer from 10 μM stock, 0.2 μl reverse primer from 10 μM stock and 10 μl probe GoTaq qPCR master mix [Promega] for a final reaction volume of 20 μl). Plates were sealed with Titer Stick HC Film (Biolabs). Reactions were performed using an AB Biosystems StepOnePlus™ System using the following program: 1 cycle of 94 °C for 5 min; 40 cycles of 94 °C for 20 s, 55 °C for 20 s, and 72 °C for 30 s, followed by 1 cycle of 40 °C for 30 s. Absolute copy numbers per gram of feces were calculated based on standard curve values obtained for respective bacterial and fungal analyses (ranging from 10 to 1 × 10^5^ copies/reaction). The Ct value could not be estimated with <10 copies from bacteria or fungi, and, therefore, the detection limit was set to 10 copies/reaction, which corresponded to 10^6^ copies/g feces of both types of microorganism. A melting curve analysis was performed after amplification to distinguish the targeted PCR products from nontargeted ones. The melting curve was obtained by slow heating from 60 to 95 °C with continuous fluorescence collection. To confirm the specificity of the primers used in this study, DNA was extracted from 26 fungal species (Supplementary Table 1), and 5 ng of DNA of each species was subjected to qPCR. Although Candida_albicans_138Fw/234Rv was found to cross-react to *C. dubliniensis* as well as *C. albicans*, Ctro/UNI2 was found to be specific to the target species. PCFw/PCRv reacted to type strain of *P. copri* and did not cross-react to non-targeted bacterial species, however, it also did not react to several *P. copri* strains which were isolated from fecal samples in this study (Supplementary Table 1).

### Fluorescence in situ hybridization

The colons were isolated from mice at day 26 after colonization of *C. albicans* and fixed in methanol-Carnoy’s fixative (60% methanol, 30% chloroform, and 10% acetic acid). Paraffin-embedded sections (5 μm) were then dewaxed and hydrated. Probe Cy3-conjugated Dual 1249 (5′-GCCAAGGCTTATACTCGCT-3′)^60^ and Cy5-conjugated PRV392 (5′-GCACGCTACTTGGCTGG-3′)^61^ were used for detection of *Candida* and *Prevotella*, respectively. To evaluate the number of *Candida* and *Prevotella* in feces, PFA-fixed fecal suspensions were spread on 10 mm square compartments of a slide glass and dried up at 40 °C for 1 h. The sections were incubated with 1 µg of the respective probes in 200 µl hybridization buffer (750 mM NaCl, 100 mM Tris-HCl [pH 7.4], 5 mM EDTA, 0.01% bovine serum albumin, 10% dextran sulfate) at 40 °C for 16 h. The sections were thoroughly rinsed using washing buffer (50 mM NaCl, 4 mM Tris-HCl [pH 7.4], 0.02 mM EDTA), at 45 °C for 20 min and counterstained with 4′,6-diamidino-2-phenylindole (DAPI) (Vector Laboratories). Then, the sections were examined using a confocal microscope (FV1000-D; Olympus). *C. albicans* and *P. copri* colonization were recorded at three points along the length of the colon (proximal, middle, and distal), in each mouse from each group. The numbers of *Candida* and *Prevotella* in square regions (200 µm × 200 µm and 40 µm × 40 µm, respectively) were counted and the total number of each microbe in 1 g of feces was calculated.

## Supplementary information

Supplemental Table and Figures

Reporting-summary

## Data Availability

All the sequences obtained by bacterial and fungal analyses have been deposited in DRA at DDBJ (https://www.ddbj.nig.ac.jp/index-e.html) with accession number DRA007592.
