# Supplementary material for: Comparison of Japanese and Indian intestinal microbiota shows diet-dependent interaction between bacteria and fungi
Source: NPJ Biofilms Microbiomes. 2019 Dec 20;5:37. doi: 10.1038/s41522-019-0110-9 (PMC6925221; doi:10.1038/s41522-019-0110-9)

Supplementary Table1 Specificity check of *Candida* and *Prevotella copri* primers.

Microorganism	Strain No.	Candida_albicans _138Fw/234Rv	Ctro/UNI2	PCFw/PCRv
Candida albicans	IFM 40009	+	-	NT[1]
Candida dubliniensis	IFM 48313	+	-	NT
Candida tropicalis	IFM 61949	-	+	NT
Candida glabrata	IFM 5768	-	-	NT
Aspergillus fumigatus	IFM 54229	-	-	NT
Aspergillus flavus	IFM 61916	-	-	NT
Aspergillus niger	IFM 62238	-	-	NT
Aspergillus terreus	IFM 61604	-	-	NT
Acremonium alternatum	IFM 61908	-	-	NT
Cladosporium cladospoioides	IFM 62110	-	-	NT
Cladosporium herbarum	IFM 57770	-	-	NT
Cryptococcus neoformans	IFM 46660	-	-	NT
Cryptococcus aureus	IFM 50258	-	-	NT
Fusarium oxysporum	IFM 62224	-	-	NT
Fusarium solani	IFM 62065	-	-	NT
Penicillium oxalicum	IFM 49446	-	-	NT
Penicillium digitatum	IFM 60598	-	-	NT
Penicillium chrysogenum	IFM 61632	-	-	NT
Penicillium citrinum	IFM 62178	-	-	NT
Tricoderma viride	IFM 51186	-	-	NT
Tricoderma koningii	IFM 51050	-	-	NT
Mucor ramosissimus	IFM 47659	-	-	NT
Rhizopus oryzae	IFM 47055	-	-	NT
Saccharomyces cervisiae	IFM 40022	-	-	NT
Rhodotorula mucilaginosa	IFM 40060	-	-	NT
Rhodosporidium babjevae	IFM 48570	-	-	NT
Prevotella copri	JCM 13464	NT	NT	+
Prevotella copri	Indian #31	NT	NT	+
Prevotella copri	Indian #38	NT	NT	+
Prevotella copri	Indian #7	NT	NT	-
Prevotella copri	Japanese F37	NT	NT	+
Prevotella copri	Japanese N001-8-1	NT	NT	-
Prevotella copri	Japanese N0165	NT	NT	-
Prevotella corporis	JCM 8529	NT	NT	-
Aeromonas hydrophila	NBRC 3820	NT	NT	-
Bacteroides fragilis	JCM 11019	NT	NT	-
Bacteroides sartorii	JCM 17136	NT	NT	-
Bacteroides vulgatus	JCM 5826	NT	NT	-
Bifidobacterium longum	JCM 1217	NT	NT	-
Blautia producta	JCM 1471	NT	NT	-
Citrobacter koseri	JCM 1658	NT	NT	-
Collinsella intestinalis	JCM 10643	NT	NT	-
Escherichia coli	JCM 1649	NT	NT	-
Eubacterium dolichum	JCM 10413	NT	NT	-
Fusobacterium varium	JCM 6320	NT	NT	-
Lactobacillus acidophilus	JCM 1132	NT	NT	-
Lactobacillus gasseri	JCM 1131	NT	NT	-

[1] NT, Not tested.

Supplementary Figure 1

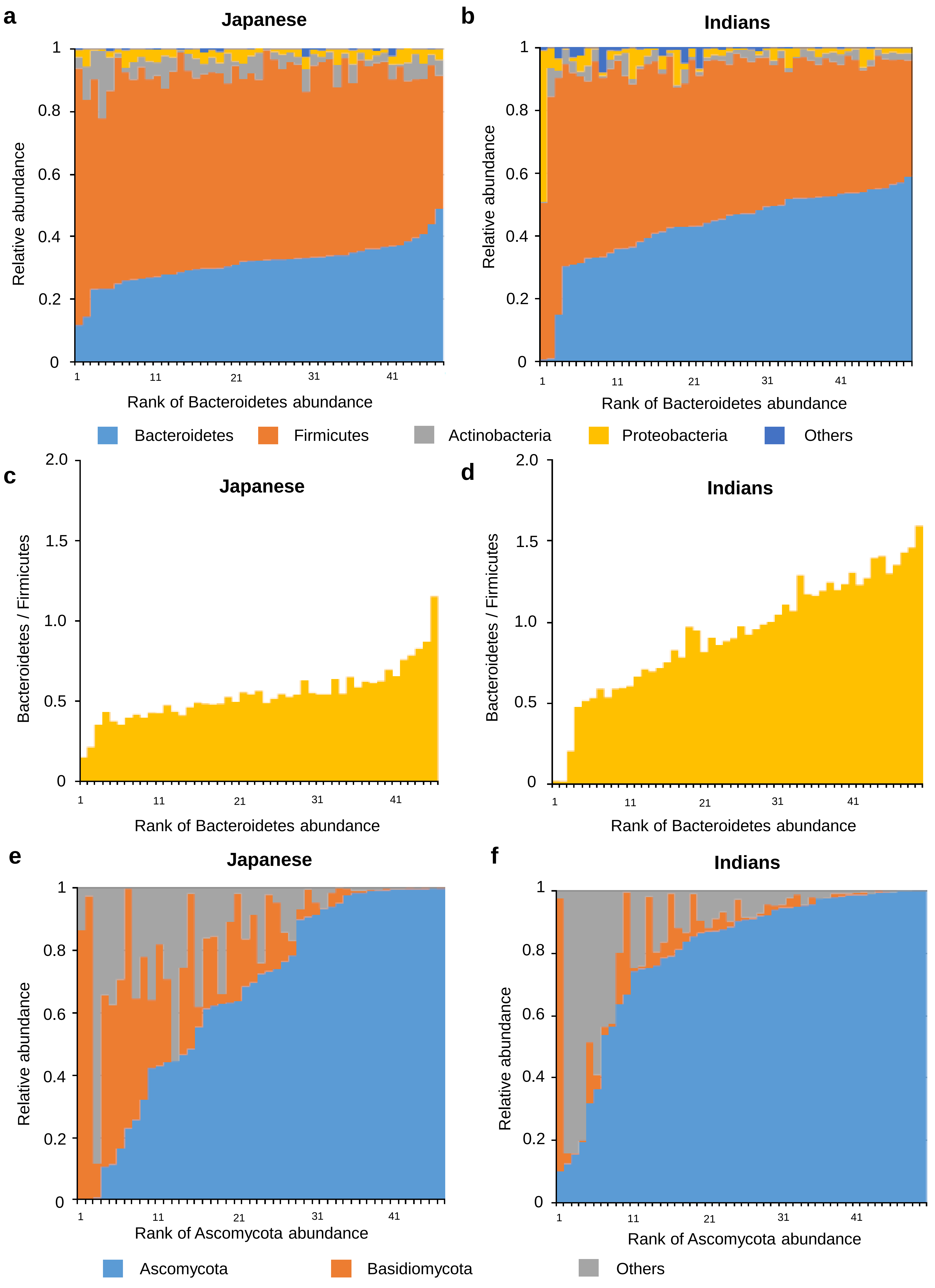

Supplementary Figure 2

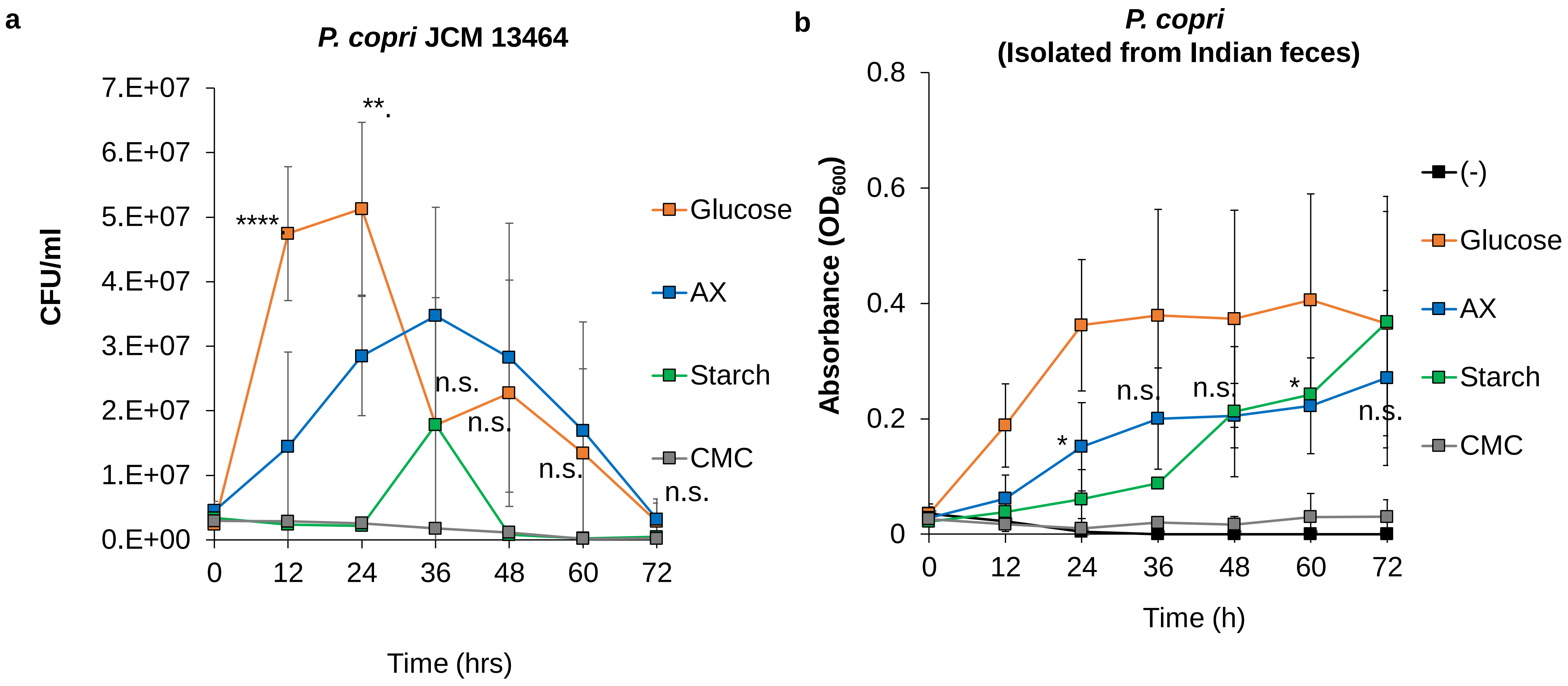

Supplementary Figure 3

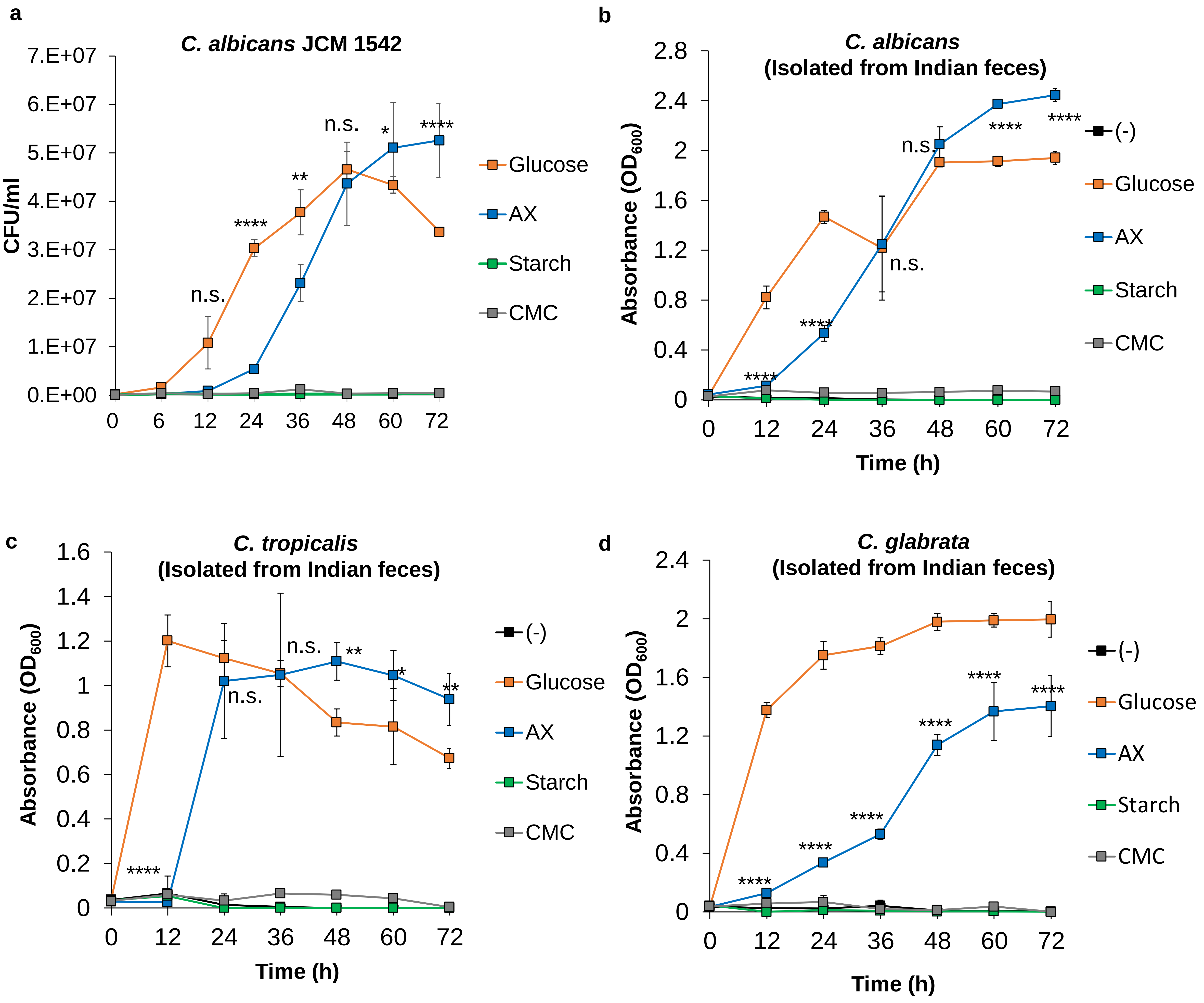

Supplementary Figure 4

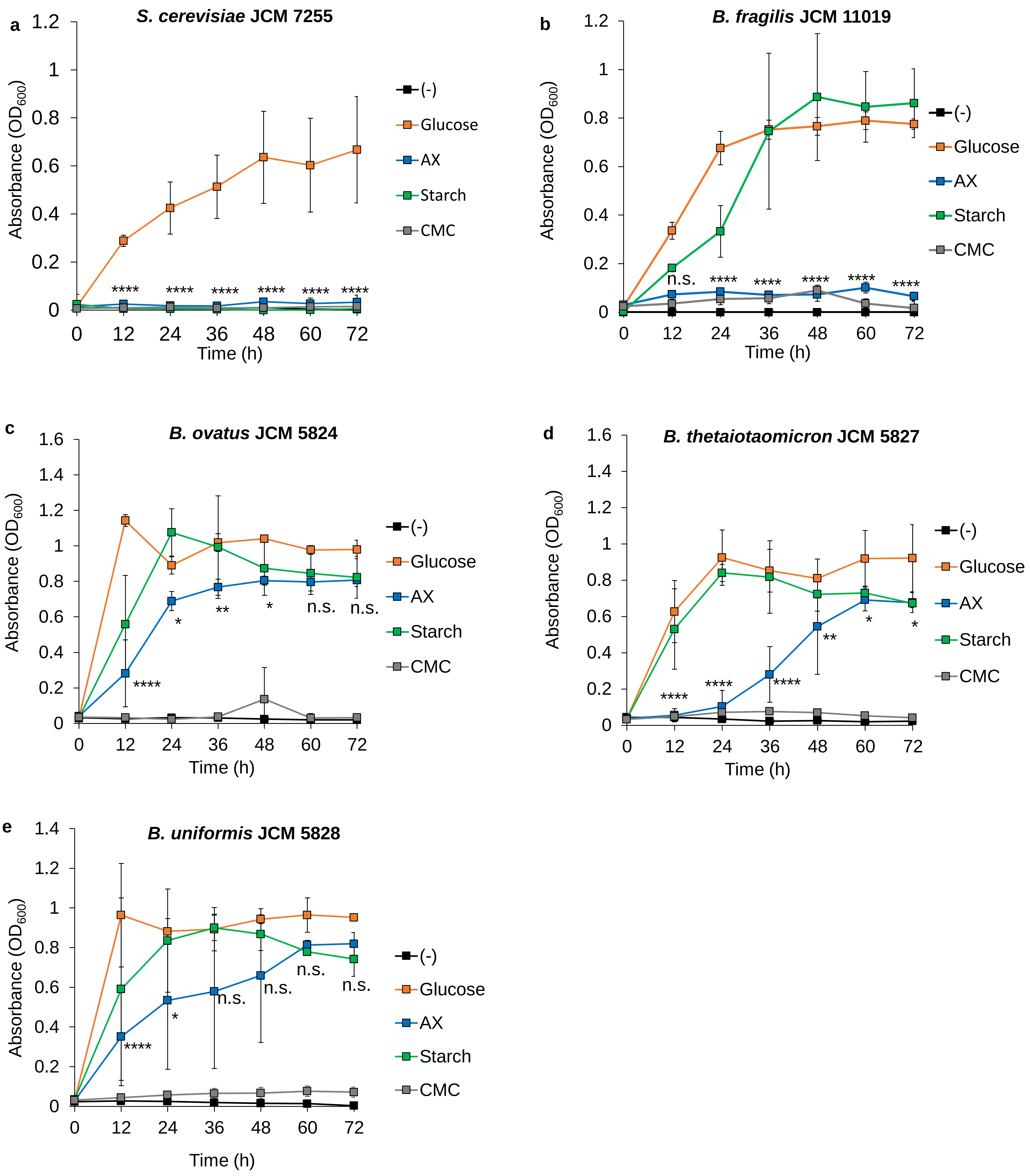

Supplementary Figure 5

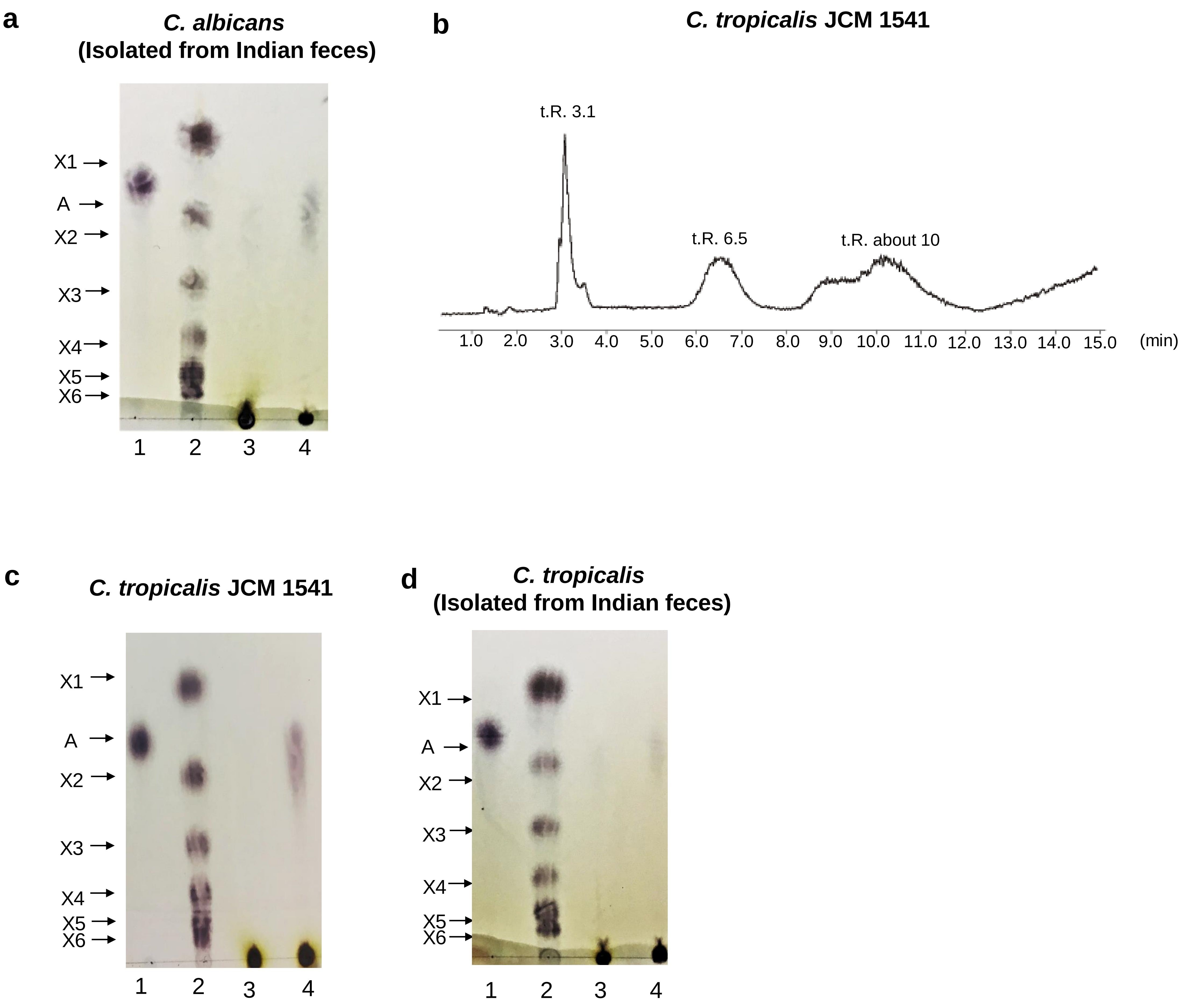

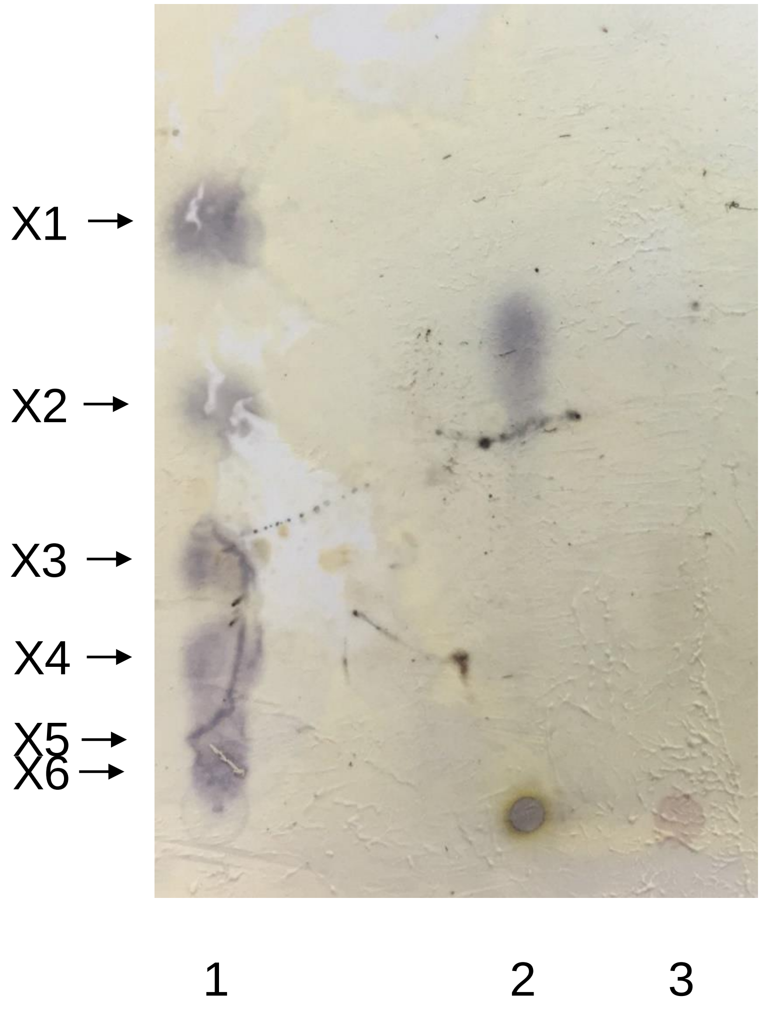
X1
X2
X3
X4
X5
X6
1
2
3

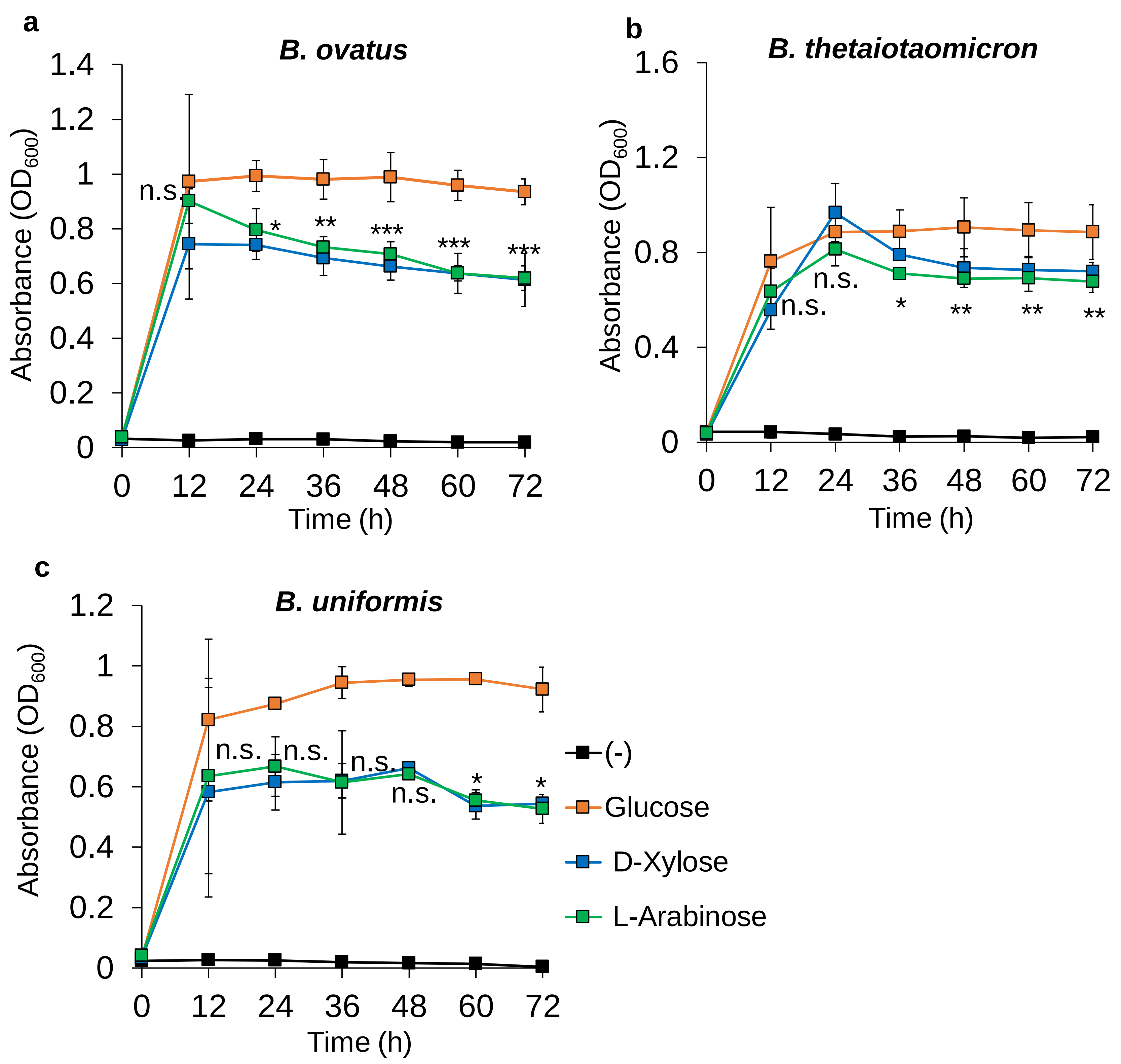
a
B. ovatus
Absorbance (OD600)
1.4
1.2
1
0.8
0.6
0.4
0.2
0
n.s.
*
**
***
***
***
0
12
24
36
48
60
72
Time (h)
b
B. thetaiotaomicron
Absorbance (OD600)
1.6
1.2
0.8
0.4
0
n.s.
n.s.
*
**
**
**
Time (h)
c
B. uniformis
1.2
1
0.8
0.6
0.4
0.2
0
n.s.
n.s.
n.s.
n.s.
*
*
Time (h)
(-)
Glucose
D-Xylose
L-Arabinose

Supplementary Figure 8

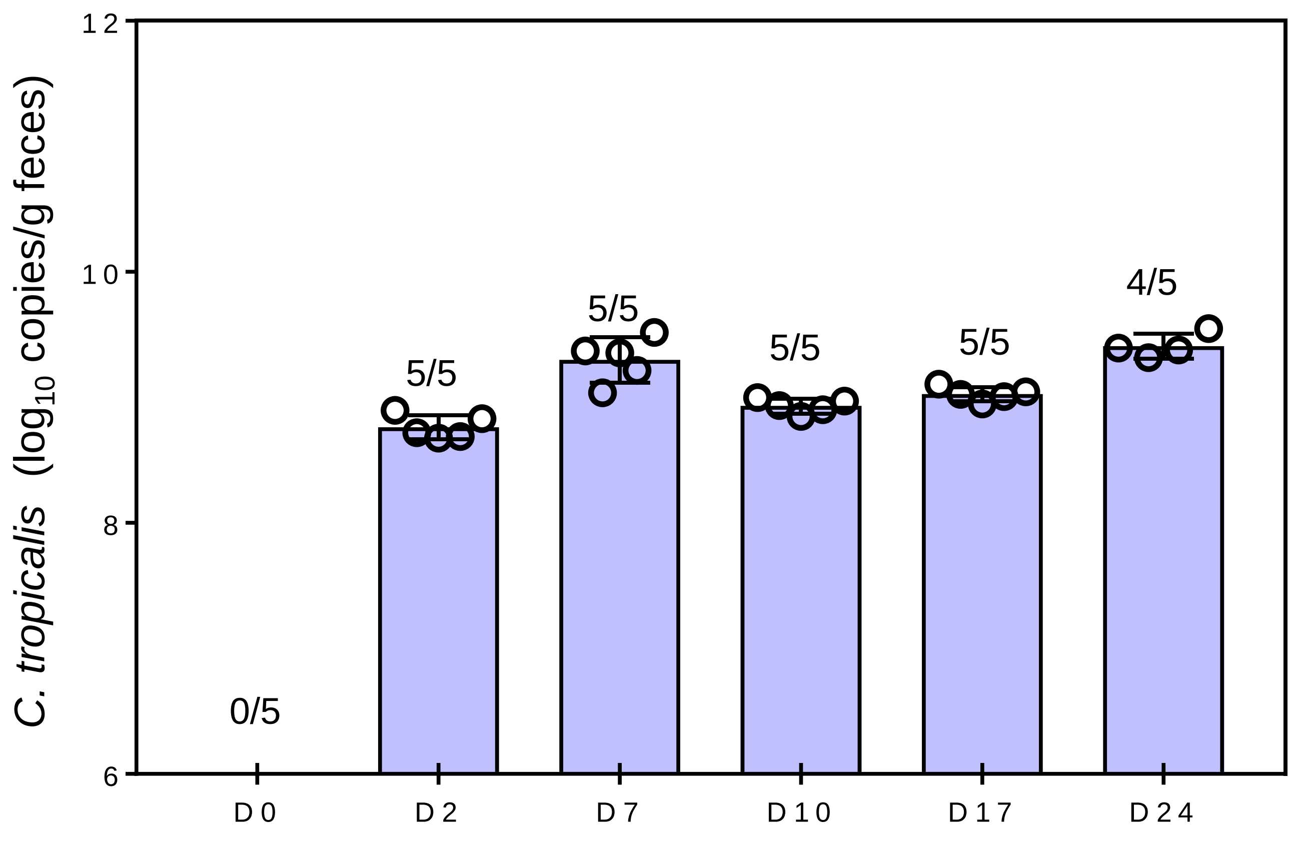

Supplementary Figure 9

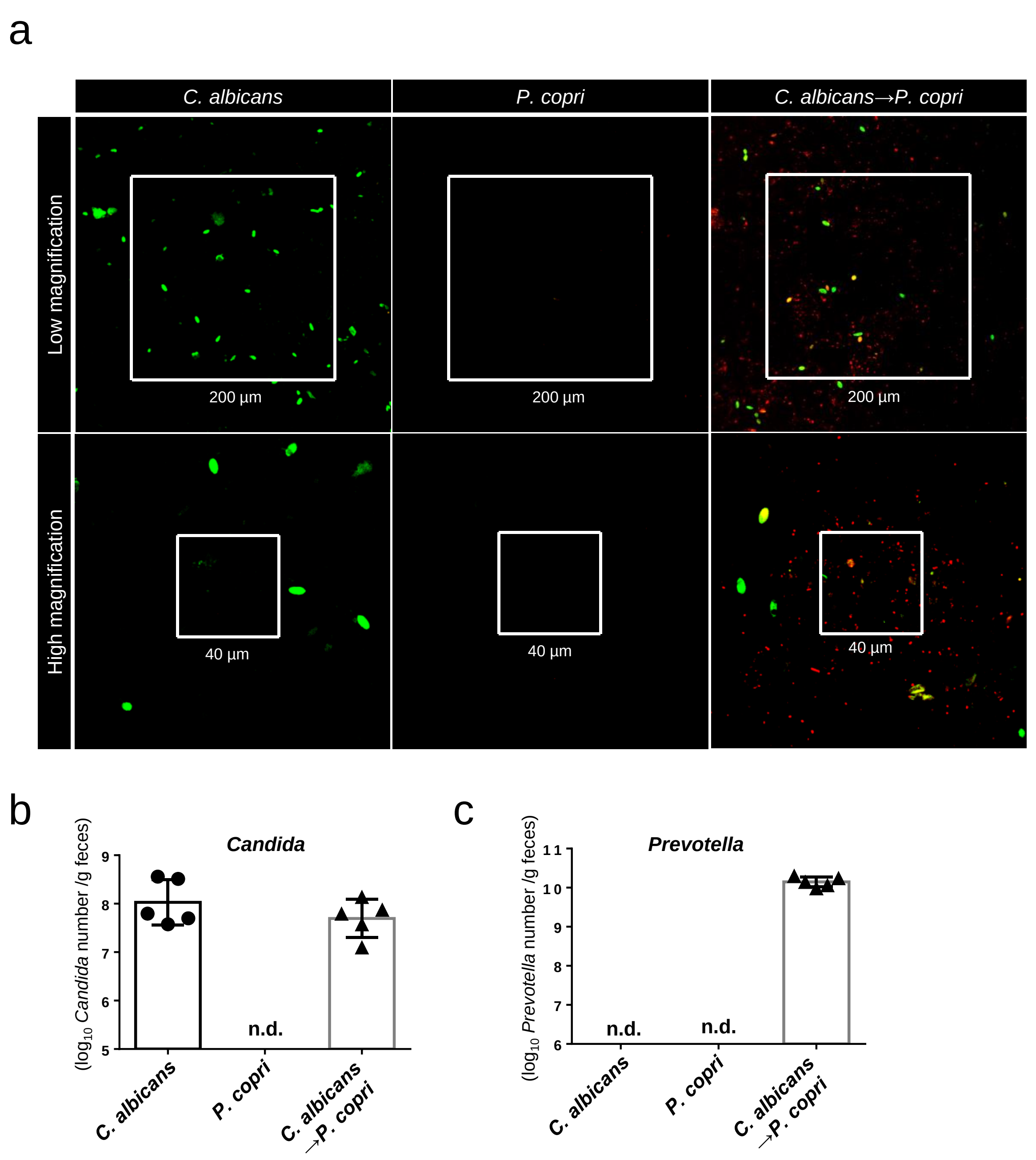

Supplement: Supplementary file 1 — Supplemental Table and Figures [file 41522_2019_110_MOESM1_ESM.pdf]
